# PeptideShaker Online: A User-Friendly Web-Based Framework for the Identification of Mass Spectrometry-Based Proteomics Data

**DOI:** 10.1021/acs.jproteome.1c00678

**Published:** 2021-10-28

**Authors:** Yehia Mokhtar Farag, Carlos Horro, Marc Vaudel, Harald Barsnes

**Affiliations:** † Proteomics Unit, Department of Biomedicine, University of Bergen, 5020 Bergen, Norway; ‡ Computational Biology Unit, Department of Informatics, University of Bergen, 5008 Bergen, Norway; § Department of Clinical Sciences, University of Bergen, 5020 Bergen, Norway

**Keywords:** mass spectrometry, data processing, Galaxy, interaction, visualization

## Abstract

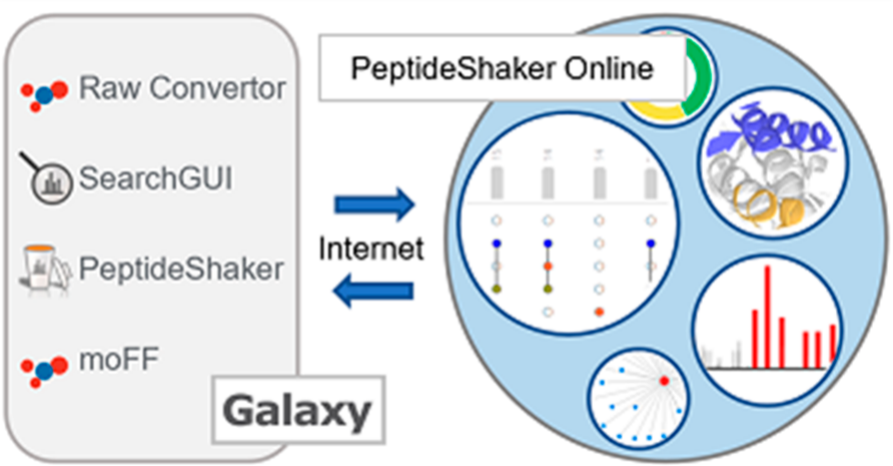

Mass spectrometry-based proteomics is a high-throughput technology generating ever-larger amounts of data per project. However, storing, processing, and interpreting these data can be a challenge. A key element in simplifying this process is the development of interactive frameworks focusing on visualization that can greatly simplify both the interpretation of data and the generation of new knowledge. Here we present PeptideShaker Online, a user-friendly web-based framework for the identification of mass spectrometry-based proteomics data, from raw file conversion to interactive visualization of the resulting data. Storage and processing of the data are performed via the versatile Galaxy platform (through SearchGUI, PeptideShaker, and moFF), while the interaction with the results happens via a locally installed web server, thus enabling researchers to process and interpret their own data without requiring advanced bioinformatics skills or direct access to compute-intensive infrastructures. The source code, additional documentation, and a fully functional demo is available at https://github.com/barsnes-group/peptide-shaker-online.

## Introduction

Mass spectrometry-based proteomics generates large amounts of data,^1^ and it is essential that the data can be processed and analyzed in such a way that the researcher generating the data can interpret its biological meaning correctly. In addition to biological knowledge, this often requires direct access to significant computational resources and advanced computational skills. The overall challenge can be split into three main categories: (i) access to computational resources; (ii) availability of user-friendly bioinformatics software; and (iii) having the biological understanding to translate the data into useful knowledge.

The first category can be addressed by high-performance computing environments that provide the required resources through powerful servers instead of the more limited personal computers,^2^ while at the same time making the stored data more portable and accessible; i.e., there is no need to download or move the data.^3^ Adding interactive visualization to such setups can help with the second category of the need for user-friendly bioinformatics software, and can also play a key role in the data processing and simplify the interpretation of the results.^4^ Interactive visualization can furthermore greatly reduce the complexity of interpretation by providing direct interaction with the data and by dividing it into distinct levels, thus enabling the biological researcher to focus on interpreting the data and extracting biological knowledge.^5^

One way to port bioinformatic pipelines to remote servers is to take advantage of Galaxy, a web-based scientific analysis platform including more than 5500 specialized tools, in addition to workflow support and data storage management, thus providing the required infrastructure for large-scale proteomics data analysis.^6^ Galaxy is however limited when it comes to advanced interactive visualization of the results and may not be straightforward to use for nonprogrammers.

As a response to these challenges, we here present PeptideShaker Online, a user-friendly web-based framework for the identification of mass spectrometry-based proteomics data, from raw file conversion to interactive visualization of the search results.

## Methods

PeptideShaker Online consists of two main components; a Galaxy-based backend where the data is stored and the search and data processing are performed,^7^ and a locally installed web-based frontend supporting SearchGUI^8^ search and interactive visualization of PeptideShaker^9^ projects. The data processing is done via the Galaxy platform using (i) ThermoRawFileParser^10^ for converting Thermo raw files into mzML^11^ or mgf; (ii) SearchGUI for protein identification based on ten proteomics search and de novo engines, namely OMSSA,^12^ X! Tandem,^13^ MyriMatch,^14^ MS Amanda,^15^ MS-GF+,^16^ Comet,^17^ Tide,^18^ MetaMorpheus,^19^ DirectTag,^20^ and Novor;^21^ (iii) PeptideShaker for interpretation of the peptide identification data from SearchGUI; and finally (iv) moFF for extracting MS1 intensities from the mass spectra.^22^ Spectrum input is supported as either mgf or mzML for identification, and Thermo raw files for both identification and quantification.

Vaadin 7.26 (https://vaadin.com) and Java 8 are used for the frontend implementation, and Tomcat server version 9 (https://tomcat.apache.org) is used to host the demo web application. Reactome^23^ is used for the proteoform network data, Lite-Mol^24^ for the protein 3D structures, compomics-utilities 5.0.15^25^ to produce the main spectrum charts, and Jersey 2.34 (https://eclipse-ee4j.github.io/jersey) for managing the connections between Galaxy and PeptideShaker Online.

For a complete list of libraries, the full source code, additional documentation, and step-by-step instructions on how to deploy PeptideShaker Online on your own web server, please see https://github.com/barsnes-group/peptide-shaker-online.

## Results

**Figure 1:**
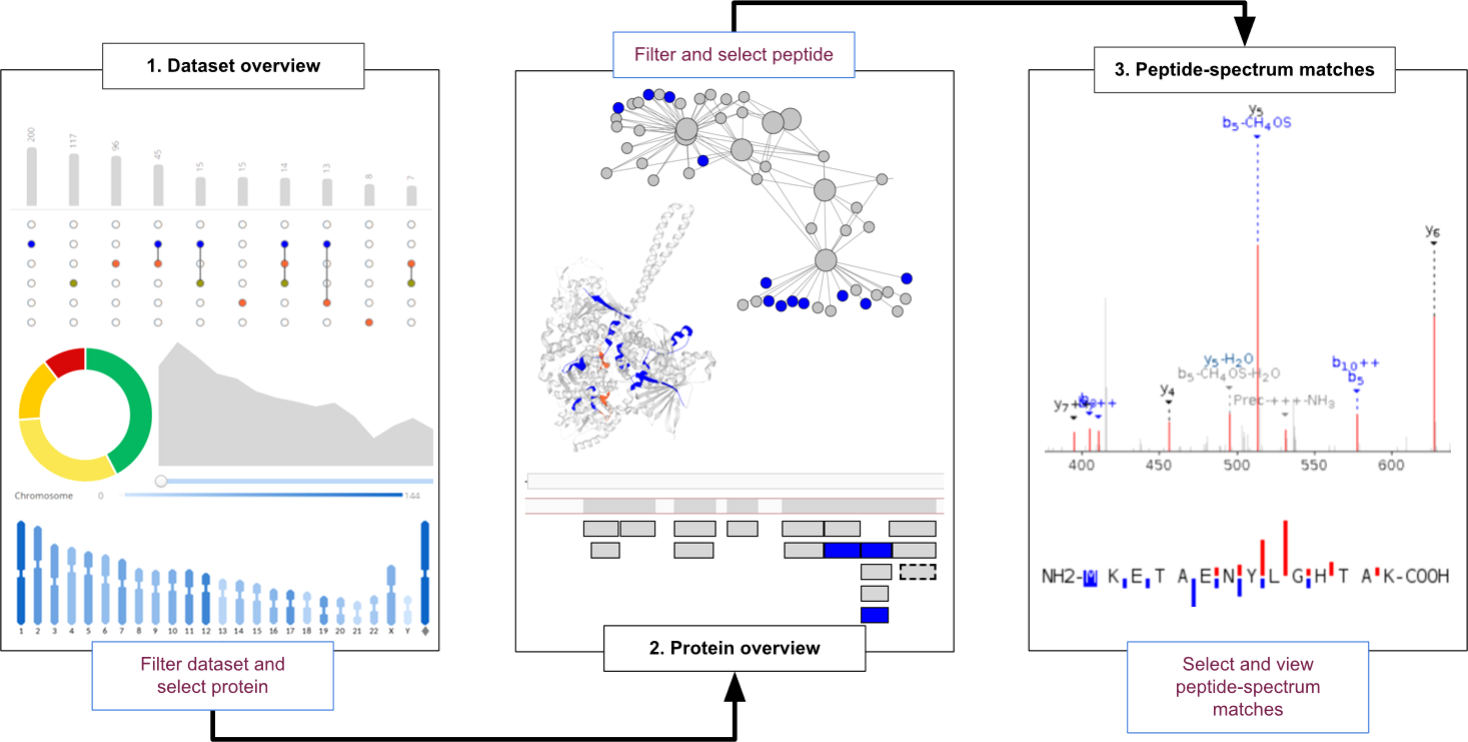
Overview of the three data set levels and the filter-and-select approach. The user filters the data set at the Data Set Overview level to find and select a protein (group) for closer inspection at the Protein Overview level. Here the user selects a peptide from the protein–peptide network or protein coverage table in order to see the peptide and spectrum details at the Peptide-Spectrum Matches level.

As a web-based interactive proteomics framework, PeptideShaker Online can be deployed in proteomics laboratories and facilities. It aims to simplify the mass spectrometry-based proteomics data identification through providing the users with an intuitive easy-to-use interface, thus removing the need for advanced programming skills or the use of command lines. Additionally, the result interpretation is enhanced through supporting coordinated interactive visualization combined with splitting the data into three separate levels: (i) Data Set Overview, (ii) Protein Overview, and (iii) Peptide-Spectrum Matches. Thus, instead of dealing with the data as one large and complex unit, the user can inspect the data at three separate levels through a filter-and-select approach (Figure 1).

**Figure 2:**
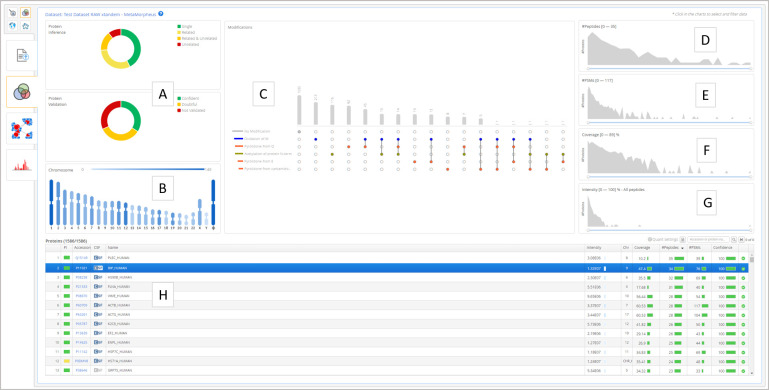
Data set overview. (A) Protein inference and validation filters, (B) chromosome filter, (C) post-translational modifications filter, (D) number of peptides filter, (E) number of peptide-spectrum filter, (F) protein coverage filter, (G) protein intensity filter, and (H) protein table with the currently filtered results.

The top level is the Data Set Overview (Figure 2), providing a summary of the currently selected data set and searching for the proteins of interest through interactive filtering of the protein identifications, including: (i) post-translational modifications; (ii) protein inference and validation categories; (iii) number of peptides and peptide-spectrum matches, in addition to protein coverage and quantification intensity; (iv) chromosome mapping; and finally, (v) a protein table showing the currently filtered proteins.

**Figure 3:**
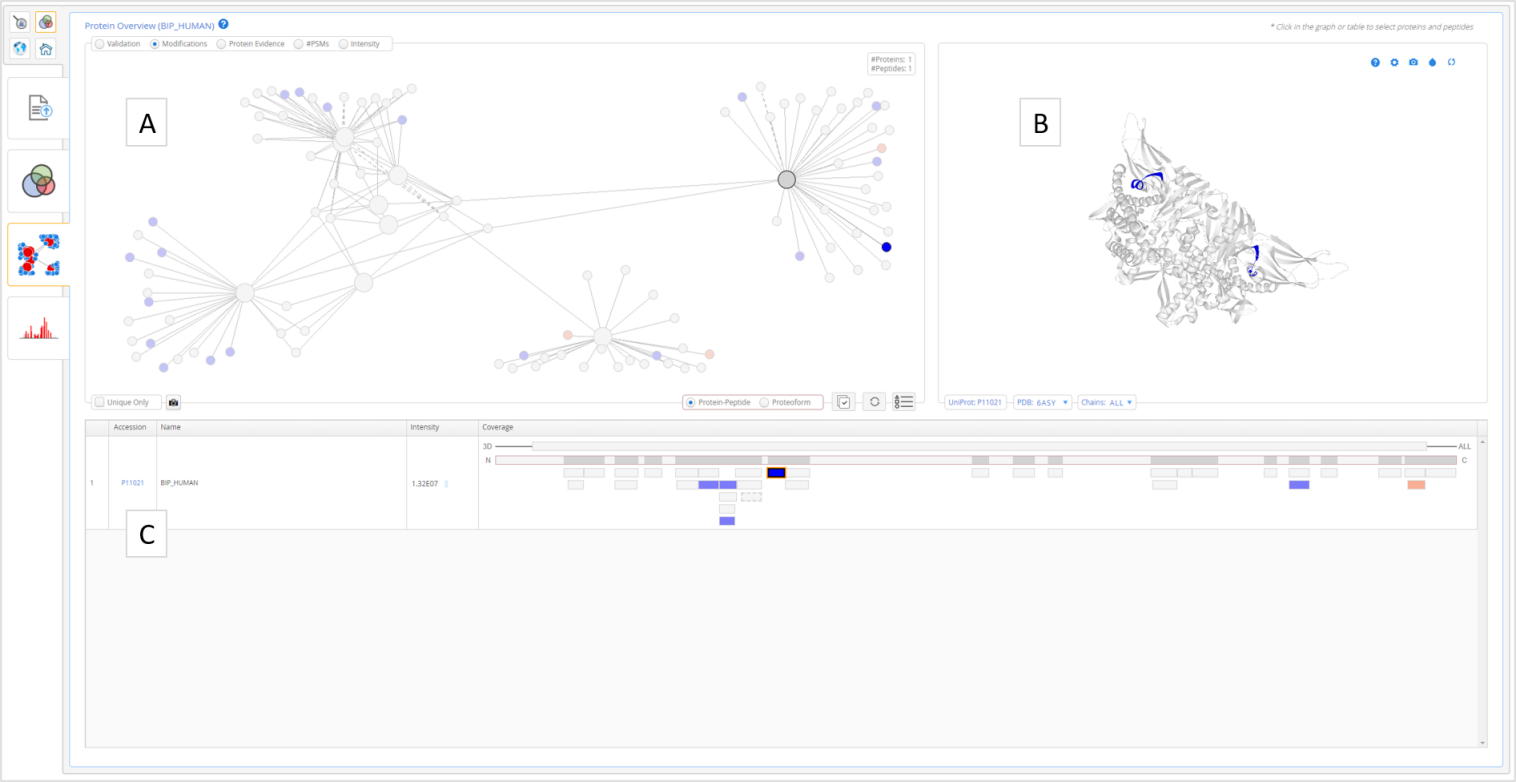
Protein overview. (A) Protein-peptide (or proteoform) network, (B) protein 3D structure, and (C) protein coverage table.

Next is the Protein Overview (Figure 3), showing the details for the currently selected protein, including: (i) a protein-peptide network (with related protein groups and peptides) or a proteoform network for the related proteoforms interactions; (ii) the protein 3D structure; and (iii) the protein coverage. For all three visualizations, the peptides are color-coded according to a user-selected property, e.g., the number of peptide-spectrum matches or the post-translational modification type.

**Figure 4:**
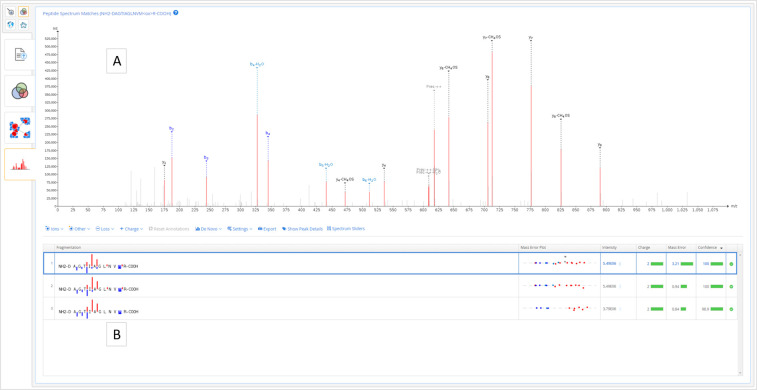
Peptide-spectrum matches. (A) Interactive spectrum viewer, and (B) peptide-spectrum matches table.

Finally, there is the Peptide-Spectrum Matches level (Figure 4), including the annotated spectrum matches for the selected peptide. Notably, the spectrum viewer is fully interactive and supports both manual and automatic de novo sequencing in addition to customizable peak annotation. This level also includes a peptide-spectrum matches table with sequence fragmentation charts and mass error plots.

Furthermore, PeptideShaker Online makes it possible for users to share their own processed results using project-specific links. Besides saving time, this feature makes the data more secure and portable given that there is no transfer of the underlying data files between the users. The users can also export the data directly as either an Excel spreadsheet or images. Finally, PeptideShaker Online also supports the uploading and visualization of locally processed data files in tab-delimited file formats, making it possible to use the framework without having to reprocess the data with the full pipeline, for example, when the desired spectrum files are not available.

To test the framework and its main features, a fully functional demo is available, which includes two processed data sets (Table 1) and supporting new searches based on the example data provided. Due to local resource limitations, the maximum number of concurrent users for the demo is set to five. Note that the demo uses a public Galaxy user key by default. When installing their own version of PeptideShaker Online, users should rather use personal Galaxy API keys to control access to the data. For information about how to set up your own PeptideShaker Online web server, please visit the project’s GitHub page: https://github.com/barsnes-group/peptide-shaker-online.

**Table 1:** Details for the Example Datasets Included in the Demo Version of PeptideShaker Online

name	spectrum format	FASTA	search parameters	search engines	id	quant
Sample 1	mzML	The reviewed sequences for human from UniProt^26^	Modifications: Oxidation of M, (variable) and Carbamidomethylation of C (fixed). Enzyme: Trypsin with max two missed cleavages. Tolerances: 10 ppm (precursors) and 0.02 Da (fragment ions).	X! Tandem, MS-GF+, OMSSA, Comet, Tide, MyriMatch, MetaMorpheus, MS Amanda, DirectTag and Novor	yes	
Sample 2	raw	The reviewed sequences for human from UniProt	Modifications: Oxidation of M, (variable) and Carbamidomethylation of C (fixed). Enzyme: Trypsin with max two missed cleavages. Tolerances: 10 ppm (precursors) and 0.02 Da (fragment ions).	X! Tandem, MS-GF+, OMSSA, Comet, Tide, MyriMatch, MetaMorpheus, MS Amanda, DirectTag and Novor	yes	yes

## Conclusion

In summary, PeptideShaker Online is a user-friendly web-based framework for the identification of mass spectrometry-based proteomics data, from raw file conversion to interactive visualization of the results. The framework is easily expandable by either including additional tools from the Galaxy platform or introducing new data visualization levels in the web-based frontend. PeptideShaker Online makes the identification of proteomics data more accessible to researchers lacking advanced computational skills, thus moving the data interpretation closer to the biologists in charge of generating the data. Furthermore, the coordinated interactive visualizations combined with splitting the data into distinct levels allows for intuitive data exploration and thus contributes to a better understanding of proteomics data and its inherent complexity.
